# Diverse myopathological features in the congenital myasthenia syndrome with GFPT1 mutation

**DOI:** 10.1002/brb3.2469

**Published:** 2022-01-03

**Authors:** Kaiyan Jiang, Yilei Zheng, Jing Lin, Xiaorong Wu, Yanyan Yu, Min Zhu, Xin Fang, Meihong Zhou, Xiaobing Li, Daojun Hong

**Affiliations:** ^1^ Department of Neurology The First Affiliated Hospital of Nanchang University Nanchang China; ^2^ Department of Medical Genetics The First Affiliated Hospital of Nanchang University Nanchang China; ^3^ Department of Ophthalmology The First Affiliated Hospital of Nanchang University Nanchang China

**Keywords:** congenital myasthenia syndrome, glutamine‐fructose‐6‐phosphate transaminase 1, myopathological changes, tubular aggregates

## Abstract

**Introduction:**

Mutations in the *GFPT1* gene are associated with a particular subtype of congenital myasthenia syndrome (CMS) called limb‐girdle myasthenia with tubular aggregates. However, not all patients show tubular aggregates in muscle biopsy, suggesting the diversity of myopathology should be further investigated.

**Methods:**

In this study, we reported two unrelated patients clinically characterized by easy fatigability, limb‐girdle muscle weakness, positive decrements of repetitive stimulation, and response to pyridostigmine. The routine examinations of myopathology were conducted. The causative gene was explored by whole‐exome screening. In addition, we summarized all *GFPT1*‐related CMS patients with muscle biopsy in the literature.

**Results:**

Pathogenic biallelic *GFPT1* mutations were identified in the two patients. In patient one, muscle biopsy indicated vacuolar myopathic changes and atypical pathological changes of myofibrillar myopathy characterized by desmin deposits, Z‐disc disorganization, and electronic dense granulofilamentous aggregation. In patient two, muscle biopsy showed typical myopathy with tubular aggregates. Among the 51 reported *GFPT1*‐related CMS patients with muscle biopsy, most of them showed tubular aggregates myopathy, while rimmed vacuolar myopathy, autophagic vacuolar myopathy, mitochondria‐like myopathy, neurogenic myopathy, and unspecific myopathic changes were also observed in some patients. These extra‐synaptic pathological changes might be associated with GFPT1‐deficiency hypoglycosylation and altered function of muscle‐specific glycoproteins, as well as partly responsible for the permanent muscle weakness and resistance to acetylcholinesterase inhibitor therapy.

**Conclusions:**

Most patients with *GFPT1*‐related CMS had tubular aggregates in the muscle biopsy, but some patients could show great diversities of the pathological change. The myopathological findings might be a biomarker to predict the prognosis of the disease.

## 1 INTRODUCTION

Congenital myasthenia syndrome (CMS) includes a large group of rare inherited endplate myopathies characterized by dysfunctions of neuromuscular junction transmission due to genetic defects (Engel et al., 2015; Finsterer, 2019). CMS shows great clinical and genetic heterogeneities characterized by abnormal fatigability, transient or permanent muscle weakness with varied age of onset. The main inheritance pattern of this disease is autosomal recessive, but a small part is inherited in autosomal dominant mode. There are at least 32 kinds of genes that have been identified in CMSs, while the number is still being updated (Iyadurai, 2020). Mutations in *CHRNA1*, *CHRNB1*, *CHRND*, or *CHRNE* are the most causative genes accounting for more than 30% of the cases, while mutations in *RAPSN*, *COLQ*, and *DOK7* involve about 10% to 15% of the cases, and *GFPT1* is accountable to approximately 3% of the cases (Engel et al., 2015; Finsterer, 2019).

Among the various types of CMS, the limb‐girdle form is characterized by a muscle weakness and fatigability predominant in proximal muscles with minor or no involvement of ocular, facial, and bulbar muscles (Belaya et al., 2012). Mutations in the glutamine‐fructose‐6‐phosphate transaminase 1 (GFPT1, Online Mendelian Inheritance in Man [OMIM]:138292) gene encoding a ubiquitous enzyme for biosynthesis pathway of protein glycosylation are responsible for a specific CMS subtype called limb‐girdle myasthenia with tubular aggregates (OMIM:610542) (Huh et al., 2012; Senderek et al., 2011). Although genetic screening may be conveniently available for these patients through next‐generation sequence (NGS), muscle biopsy is typically the first assessment conducted in these CMS patients who predominantly present with limb‐girdle muscle weakness. Considering that some subtypes of CMS may be treatable genetic diseases, it is very important to make a timely diagnosis as early as possible (Farmakidis et al., 2018). Therefore, accurate identification of the various myopathological changes is very important to the diagnosis of CMS. However, not all patients show tubular aggregates in muscle biopsy (Guergueltcheva et al., 2012), suggesting a need to re‐recognize and summarize the diversity of muscle pathology in patients with CMS associated with *GFPT1* mutations.

In this study, we described two CMS patients with *GFPT1* mutations: one presented with vacuolar myopathy with myofibrillar destruction, and the other showed typical myopathy with tubular aggregates. To further explore the pathological characteristics of CMS caused by *GFPT1* mutation, we summarized the muscle pathological features in all reported *GFPT1*‐related CMS cases with muscle biopsy.

## 2 MATERIALS AND METHODS

### 2.1 Subjects

Patients with *GFPT1* mutations were recruited from our in‐home database including 15 patients with CMS between January 2016 and June 2021. The inclusion criteria of CMS included fatigable muscle weakness presenting with ptosis, ophthalmoparesis, facial and bulbar, and generalized muscle involvement; positive changes of neuromuscular junction in electrophysiological assessments; and/or causative mutations in CMS‐related genes. A battery of clinical and laboratory investigations were conducted to exclude the inflammatory, toxic, or metabolic origins. A detailed medical history was obtained from the subjects and their relatives. Information regarding age of onset, progression of disease, family history, and other clinical manifestations was collected. Electrophysiological study was performed in the nerves using a standard method with surface electrodes for stimulation and recording.

### 2.2 Ethical statement

All patients’ tissue samples were obtained after a written consent signed by each individual in compliance with the bioethics laws of China as well as the Declaration of Helsinki. The research was approved by ethics committee of the first affiliated hospital of Nanchang University.

### 2.3 Genetic screening

Genomic DNA was extracted from peripheral blood samples. The NGS was commercially supported by Running Gene Inc. (Beijing, China). In brief, targeted exon enrichment was performed using SureSelect Human All Exon V5 (Agilent Technologies). The exon‐enriched DNA libraries were subjected to paired‐end sequencing with the Hiseq 2000 platform (Illumina, Inc.). Sequence data were mapped with BWA (Li & Durbin, 2009) and SAMTOOLS (Li et al., 2009) onto the hg19 human genome as a reference. Calls with variant quality less than 20 were filtered out, and 95% of the targeted bases were covered sufficiently to pass our thresholds for calling single nucleotide polymorphisms (SNP), nonsynonymous/splice acceptor and donor site, insertions or deletions (NS/SS/InDel) variants in the dbSNP v137, ESP6500, and 1000 Genome were removed. Synonymous changes were filtered using SIFT software (http://sift.jcvi.org). Sanger sequencing with specific primers was conducted to confirm the *GFPT1* mutation in the patients and their available family members.

### 2.4 Muscle pathological examination

Muscle biopsies were performed from the right bicep or left gastrocnemius of the two cases, respectively. The muscle tissue was frozen and then cut at 8 μm sections. These sections were stained according to standard histological and enzyme histochemical procedures with hematoxylin and eosin (H&E), modified Gomori trichrome (MGT), periodic acidic Schiff (PAS), oil red O (ORO), nicotinamide adenine dinucleotide tetrazolium reductase (NADH‐TR), succinate dehydrogenase (SDH), cytochrome c oxidase (COX), nonspecific esterase (NSE), and ATPase stain. Antibodies of desmin (Abcam, ab6322, 1:100), dystrophin (Leica Biosystems, NLC‐DYS2, 1:20), dysferlin (Leica Biosystems, Ham1/7B6, 1:40) and MHC‐I (Dako, R7000, 1:200) were used to detect the distribution of protein in the muscle specimens by immunohistochemical stain.

For electron microscopy, muscle specimens were fixed in 2.5% glutaraldehyde in phosphate buffer and post‐fixed in 1% osmium tetroxide in the same buffer. Specimens were then dehydrated and embedded in Epon 812. The ultrathin sections of muscle tissue were double stained with uranyl acetate and lead citrate, and then examined with an electron microscope (JEM‐1230 JEOL Inc. Tokyo, Japan).

### 2.5 Literature review

We searched the literature in multiple databases including PubMed, EMBASE, Scopus, Web of Science, EBSCO, and Google Scholar database using the keywords “congenital myasthenia syndrome” and “*GFPT1* gene.” All included cases were required to have muscle biopsy, then the clinical characteristics, laboratory results, treatments, complications and outcomes of all patients were summarized and reanalyzed.

## 3 RESULTS

### 3.1 Clinical features

#### 3.1.1 Patient one

The patient was a 37‐year‐old man who had limb weakness for more than 30 years. At age 5, the patient has noticed poor ability in walking and running compared to his peers. At age 15, he had difficulty in standing up after squatting, and frequently falling down. The symptom of muscle weakness was better in the morning, but worse in the evening. He was diagnosed with myasthenia gravis. Corticosteroid was initially administered, but no efficacy was observed. Afterward, he showed some responses to pyridostigmine, while the muscle weakness gradually progressed to walking difficulty and bath inability. His family history was unremarkable.

Physical examination on admission revealed symmetrical limb weakness without facial, bulbar, neck, and respiratory muscle involvement. Muscle strength (Medical Research Council, MRC) was 4 grade in the proximal upper limbs, 5‐ grade in the distal upper limbs, 3 grade in the proximal lower limbs, and 4 grade in the distal lower limbs. Deep tendon reflexes were decreased. Pathological reflexes were negative. No muscle atrophy or fasciculation could be observed. There was no evidence of sensory disturbance, ataxia, or autonomic dysfunction.

**FIGURE 1:**
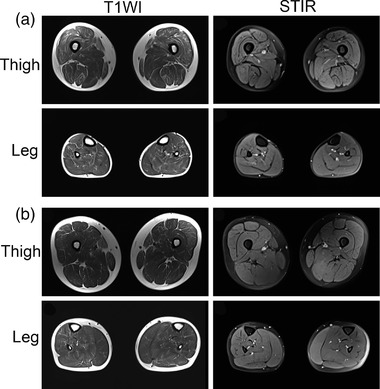
Muscle MRI changes of lower limb in patient one (a) and patient two (b) with *GFPT1*‐related CMS. Thigh level showed a slightly diffused hyperintensity on T1WI except adductor magnus and semimembranosus muscles; leg level showed a slightly diffused hyperintensity except medial gastrocnemius that simultaneously had mild high‐signal on STIR

The blood count, blood biochemistry, thyroid function, parathyroid hormone, blood acylcarnitines and urine organic acid profiles, paraneoplastic antibody spectrum, and antibodies of myasthenia gravis were all negative. Muscle MRI of thigh revealed a slightly diffused hyperintensity on T1WI except adductor magnus and semimembranosus muscles; and muscle MRI of leg also showed a slightly diffused hyperintensity except medial gastrocnemius (Figure 1a). Nerve conduction velocity (NCV) and electromyography (EMG) were not obvious abnormal. Nevertheless, repetitive nerve stimulation (RNS) at 3 Hz revealed positive decrements of compound muscular action potential (CMAP) in the deltoid.

#### 3.1.2 Patient two

The patient was a 21‐year‐old man who had limb weakness for 14 years. At age 7, he showed a poor performance in physical education class, and had a little difficulty in running and stairs climbing. Since then, he had complained of muscle fatigue and fluctuating weakness. He was diagnosed with lipid storage disease at age 12, and was given riboflavin and coenzyme Q10, but no benefits were observed. On this admission, he showed permanent weakness characterized by difficulties in climbing stairs, standing up after squatting, and combing hair.

Physical examination showed waddling gait and symmetrical proximal limb weakness without facial, bulbar, neck, and respiratory muscle involvement. Muscle strength was 3+ grade in the proximal upper limbs, 5‐ grade in the distal upper limbs, 3 grade in the proximal lower limbs, and 4+ grade in the distal lower limbs. Deep tendon reflexes could be induced. Pathological reflexes were negative. No muscle atrophy or fasciculation could be observed. No evidence of sensory disturbance, ataxia, or autonomic dysfunction was noticed.

The blood count, blood biochemistry, thyroid function, parathyroid hormone, blood acylcarnitines and urine organic acid profiles, and antibodies of myasthenia gravis were all negative. Muscle MRI of thigh revealed a mildly diffused hyperintensity on T1WI; and muscle MRI of leg also showed a slightly diffused hyperintensity except medial gastrocnemius that simultaneously had mild hyperintensity on STIR (short tau inversion recovery) (Figure 1b). NCV had no abnormality. EMG showed rapid recruitment of motor units suggestive of a myopathic pattern. In addition, RNS at 3 Hz revealed positive decrements in the deltoid and abductor digiti minimi muscle.

### 3.2 Genetic findings

**FIGURE 2:**
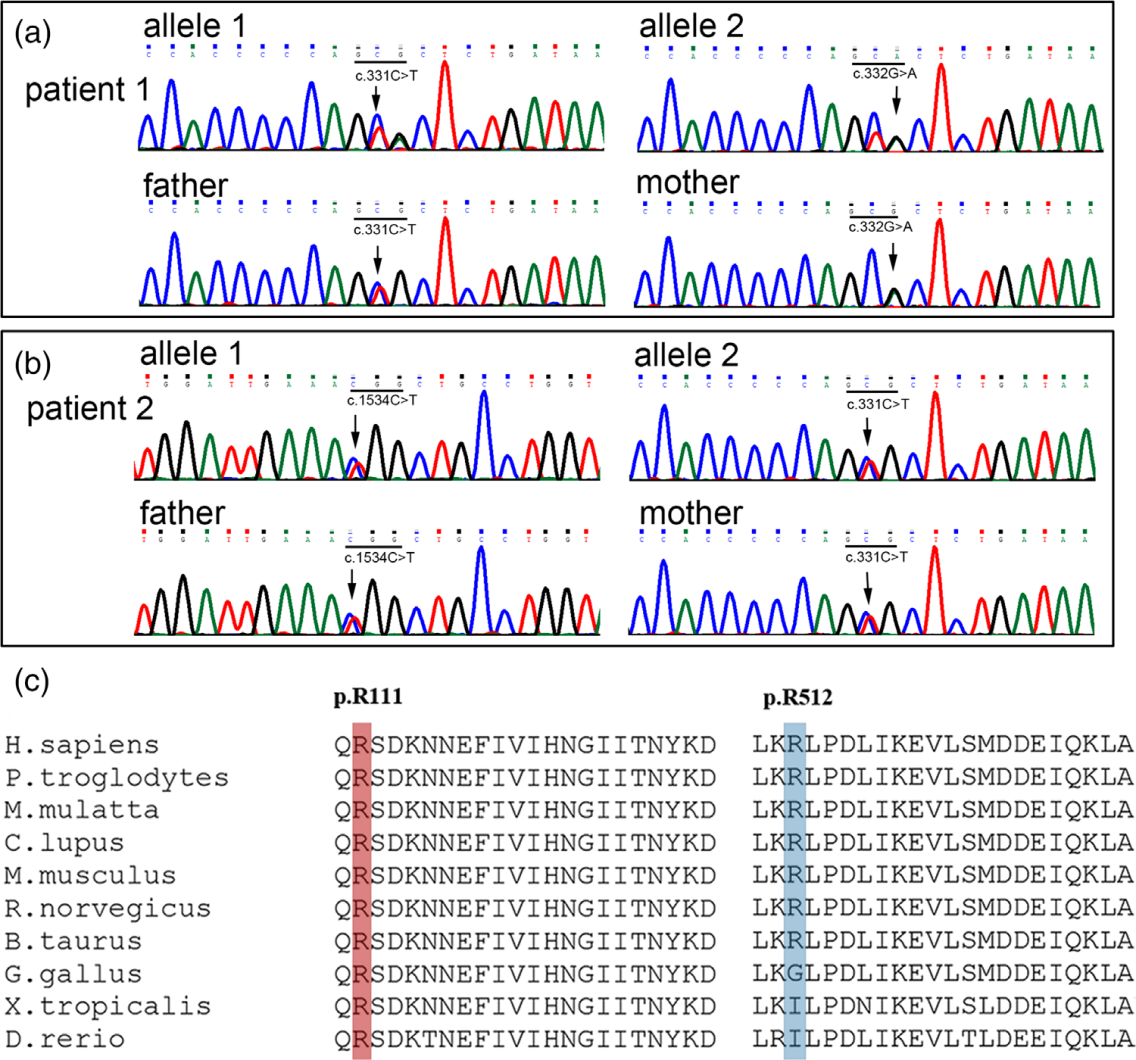
Genetic mutations in the *GFPT1* gene. Genetic sequencing disclosed compound heterozygous mutations c.331C > T and c.332G > A in patient one (a); c.331C > T and c.1534C > T in patient two (b). The variants co‐segregated with their parents. Residues arginine 111 and 512 have high evolutionary conservations (c)

Genetic sequencing disclosed compound heterozygous mutations in *GFPT1*: c.331C > T (p.R111C) and c.332G > A (p.R111H) in the patient one (Figure 2a); c.331C > T (p.R111C) and c.1534C > T (p.R512W) in the patient two (Figure 2b). The variants co‐segregated with their parents: c.332G > A was from the mother and c.331C > T was from the father; c.331C > T was from the mother and c.1534C > T was from the father. All variants have been previously reported in other patients, and had a very low allele frequency in gnomAD database (http://gnomad.broadinstitute.org, v2.1.1, Table S1). A homology search in different species demonstrated that the amino acids at residues 111 and 512 were evolutionally highly conserved, respectively (Figure 2c). The variants were predicted to be damaging by several in silico tools. The significance of variants was evaluated as pathogenic according to the American College Medical Genetics and Genomics (ACMG) criteria (Li et al., 2017). No causative mutations associated with other CMS or myopathies were found in the genetic screening.

### 3.3 Muscle pathological changes

**FIGURE 3:**
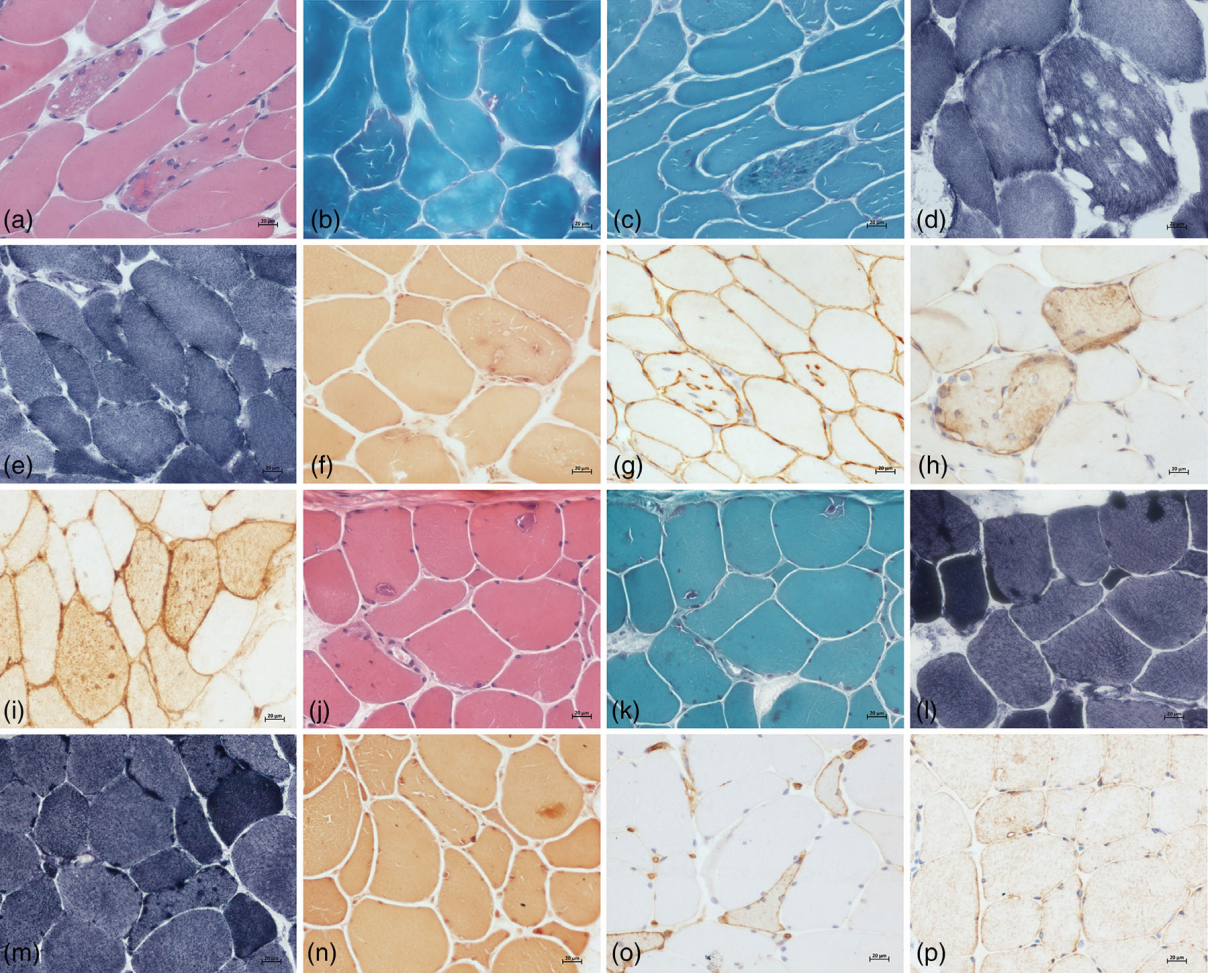
The myopathological changes in the two patients. Muscle biopsy in patient one showed multiple small vacuoles on HE stain (a), a few rimmed vacuoles (b) on MGT stain. Some fibers with small vacuoles had dark aggregations on MGT stain (c), negative to NADH stain (d,e), but positive to NSE (f), dystrophin (g), desmin (h), and MHC‐I (i). The muscle biopsy in patient two revealed tubular aggregates (j), which were dark on MGT (k), NADH (l,m), and NSE (n) stain. Some fibers had an immuno‐reactivity to MHC‐I (o), but not to desmin (p)

The myopathological changes of patient one showed an appearance of multiple small vacuoles (Figure 3a) and a few rimmed vacuoles (Figure 3b) in some fibers, accompanied with variation of fiber size, central nuclei, fiber splitting and mild interstitial proliferation. Some fibers with small vacuoles had dark aggregations on MGT stain (Figure 3c). The small vacuoles were negative to ORO, PAS, and NADH stain (Figure 3d,e), but some affected fibers were positive to NSE (Figure 3f), dystrophin (Figure 3g), desmin (Figure 3h), dysferlin, and MHC‐I (Figure 3i). On the other side, the muscle pathological features of patient two revealed tubular aggregates myopathy characterized by multiple basophilic materials deposition, variation of fiber size, central nuclei, and mild interstitial proliferation (Figure 3j). The affected fibers with tubular aggregates also showed abnormal depositions on MGT (Figure 3k), NADH (Figure 3l,m), and NSE (Figure 3n) stain. Some fibers had an immuno‐reactivity to MHC‐I (Figure 3o), but absence of desmin (Figure 3p) or other proteins aggregation.

**FIGURE 4:**
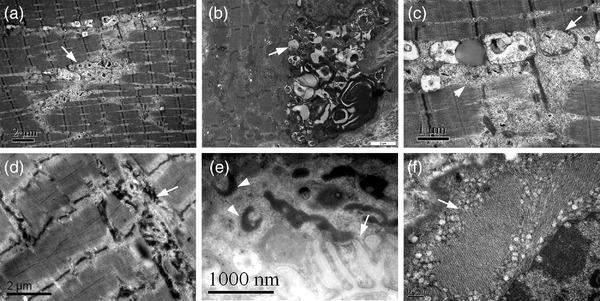
Muscle ultrastructural changes in the two patients. The muscle fibers harbored many degenerating vacuoles (a, arrow) and autophagic vacuoles (b, arrow) with myeloid bodies, vacuolated mitochondria (c, arrow), and bizarre debris (c, arrow head). Some fibers showed Z line disturbance and electronic dense granulofilamentous deposits between the myofibrils (d, arrow). Some endplates appeared reduced junctional folds (e, arrow) with ring‐like electronic dense materials (e, arrow head). Ultrastructural examination of patient two showed multiple tubular aggregates (f, arrow)

Ultrastructural examination of patient one revealed that numerous fibers harbored dilated and degenerating vesicular profiles (Figure 4a) in which were filled with autophagic vacuoles (Figure 4b), pleomorphic myeloid bodies, vacuolated mitochondria, lipofuscin granules, and bizarre debris (Figure 4c). Some fibers showed disorganization of myofibrillar structure with Z line disturbance, and some electronic dense granulofilamentous deposits under the sarcolemma and between the myofibrils (Figure 4d). In addition, some endplates appeared reduced and poorly developed junctional folds with electronic dense materials (Figure 4e). Ultrastructural examination of patient two showed local destructions of myofibrillar structure with multiple tubular aggregates (Figure 4f).

### 3.4 Response to therapy

The patient one has been taking pyridostigmine (180 mg/day) since the age of 15. The medicine worked well at first 15 years while the response became less pronounced gradually. After a definite diagnosis, he was prescribed salbutamol (6 mg/day) and fluoxetine (20 mg/day), but his symptoms showed no significant alleviation. After joint prescription to patient two of pyridostigmine (180 mg/day) and albuterol (6 mg/day), his symptoms of muscular weakness improved considerably.

### 3.5 Muscle pathological review

We summarized all reported cases of *GFPT1*‐related CMS in the past 10 years from 2011 to the present. A total of 77 patients with clinical details were reviewed (Table S2), of which 51 patients with muscle biopsy were summarized (Aharoni et al., 2017; Bauché et al., 2017; Guergueltcheva et al., 2012; Helman et al., 2019; Huh et al., 2012; Luo et al., 2019; Ma et al., 2021; Maselli et al., 2014; Matsumoto et al., 2019; Natera‐De Benito et al., 2017; O'grady et al., 2016; Prior & Ghosh, 2021; Selcen et al., 2013; Selvam et al., 2018; Senderek et al., 2011; Szelinger et al., 2020; Yiş et al., 2017; Zhao et al., 2021). The first symptoms were noted in the first decade of life in 42 of 51 patients (range from 0 to 19, median 6 years old). Besides apneic spells and survival crisis in a few patients at birth, most of them started with muscle weakness, fatigue or frequent falls due to the involvement of proximal limbs. All 51 patients showed limb‐girdle weakness, 26 (51.0%) had distal muscle weakness, 6 (11.8%) had neck weakness, 6 (11.8%) had respiratory muscle involvement, 5 (9.8%) had bulbar paralysis, and only 2 (3.9%) patients had slight ptosis.

Muscle biopsies revealed tubular aggregates in most patients, while some showed multiple pathological features (Table 1): 36 (70.6%) patients showed pure tubular aggregates; 10 (19.6%) patients presented with unspecific or mild myopathy changes but tubular aggregates accompanied in 6 patients; rimmed vacuoles occurred in 4 (7.8%) cases but simultaneously with tubular aggregates; ragged red fibers were found in 4 (7.8%) cases; neurogenic features were presented in 3 (5.9%) cases; 2 (3.9%) patients showed mild necrotizing myopathy with extensive autophagic vacuolar pathology; and 2 (3.9%) patients showed a dystrophic pattern.

**TABLE 1:** The clinical and pathological summarization of GFPT1‐related CMS patients with muscle biopsy

					Muscle biopsy findings	
References	Patient	Sex/AAO/AAD/ethnic	Clinical features	GFPT1 mutations	Light microscope	Electron microscope
Guergueltcheva 2012	1	M/6/31/Iranian	Fatigue, fluctuating LGM, distal involvement	p.D348Y (homo)	TAs, type 1 fibre predominance, atrophy fibers	ND
Guergueltcheva 2012	2	F6/26/Turk	Fluctuating LGM, fatigue, pain	p.W240X (homo)	TAs, type 2 fibers predominance, chronic myopathy	ND
Guergueltcheva 2012	3	NA/6/23‐35/Libyan	LGM, fatigue	p.R111C (homo)	Small TAs	TAs
Guergueltcheva 2012	4	M/14/55/Spanish	LGM	p.M492T; c.*22C > A	TAs, RRF, mild myopathic changes, type 1 fibre predominance	TAs
Guergueltcheva 2012	5	M/10/50/Spanish	Fluctuating LGM, falls	p.M492T; c.*22C > A	TAs, RRF, unspecific myopathic changes, type 1 fibre predominance	TAs
Guergueltcheva 2012	6	M/5/16/German	Fluctuating LGM	p.D43V; p.I121T	TAs, unspecific myopathic changes	ND
Guergueltcheva 2012	7	M/8/23/British	LGM, facial and distal muscle involvement	p.R385H; p.R434H	TAs; vacuoles, denervation changes	ND
Guergueltcheva 2012	8	M/6/37/British	Fluctuating LGM, distal limb involvement	p.T15M; p.R496W	TAs	ND
Guergueltcheva 2012	9	F/13/26/German	Fluctuating LGM, fatigue	p.V199F; c.*22 > A	TAs	ND
Guergueltcheva 2012	10	F/1/7/Senegalese	Fluctuating LGM	p.R512W (homo)	TAs, uneven oxidative staining, mitochondria accumulation	ND
Guergueltcheva 2012	11	M/7/19/Spanish	LGM	p.M491T (homo)	Unspecific myopathic changes	ND
Guergueltcheva 2012	12	M/1/37/Spanish	Fluctuating LGM	c.1278_1281dup; c.*22C > A	Unspecific myopathic changes	ND
Guergueltcheva 2012	13	M/10′s/39/Spanish	Fluctuating LGM	c.1278_1281dup; c.*22C > A	TAs, unspecific myopathic changes	TAs
Guergueltcheva 2012	14	M/10/55/Italian	LGM	p.T15A; c.621‐622del	TAs	TAs
Guergueltcheva 2012	15	M/7/36/Italian	LGM	UD	TAs	ND
Guergueltcheva 2012	16	M/10′s/40/Swedish	Fluctuating LGM	p.222‐223insA; p.R111C	TAs	TAs, PMS
Guergueltcheva 2012	17	F/8/9/Maltese	Fluctuating LGM, fatigability, learning difficulty	p.M491T; c.714_715insA	Size variability, uneven enzyme stain, type 2 fiber predominance	ND
Guergueltcheva 2012	18	M/7/13/Maltese	Fluctuating LGM, fatigability, learning difficulty	p.M491T; c.714_715insA	Size variability, uneven enzyme stain	ND
Huh 2012	19	M/13/15/Korean	LGM	p.E256Q; p.M499T	TAs	ND
Selcen 2013	20	M/0/16/NA	Poor cry, apneic spells, LGM, distal limb involvement	c.1700‐17 16dup17; c.*22C > A	Small TAs, RV	PMS, PPM, multiple myeloid structures
Selcen 2013	21	F/8/12/NA	LGM, distal limb involvement	p.R545P; c.*22C > A	Small TAs, type 1 fiber predominance	PMS, PPM
Selcen 2013	22	F/12/20/NA	LGM, distal limb involvement	c.606‐8A > G and c.*22C > A	Neurogenic features	PMS, PPM
Selcen 2013	23	M/19/56/NA	NA	p.D113G; p.M492T	Large TAs, small vacuoles	PMS, PPM
Selcen 2013	24	M/12/12/NA	LGM, distal limb involvement	p.R17X; c*22C > A	Neurogenic features	Normal EPs, myeloid structures
Selcen 2013	25	F/0/1 m/NA	Hypotonia, arthrogryposis, all weakness except ocular muscles	c.686‐2A > G; p.R304X	Small TAs, RV, AV, regenerating fibers, type 1 fiber preponderance	PMS, PPM, multiple autophagic vacuoles
Selcen 2013	26	M/10/18/NA	LGM, distal limb involvement	p.R111C (homo)	TAs	ND
Selcen 2013	27	F/9/64/NA	LGM, distal limb involvement	p.T350I; c.1337delA	Small TAs, RV, neurogenic features	ND
Selcen et al., 2013	28	M/4/9/NA	LGM, distal limb involvement	p.M1fsX2; p.T15M	TAs	ND
Maselli 2014	29	F/13/68/American	LGM, neck and distal limb involvement	c IVS7‐8A > G; c.*22C > A	Type I fiber predominance and type II fiber atrophy	PMS
O'Grady 2016	30	F/0/13/Australian	Congenital hypotonia, contractures, scoliosis	c.686‐2A > G; p.M358V	Dystrophic pattern	ND
Yis 2017	31	M/1/17/Turk	LGM, axial weakness	c.686‐2A > G (homo)	Dystrophic pattern	ND
Bauche 2017	32	NA/10′s/68/French	LGM, distal limb involvement	p.G39_ K75delinsE; p.R111H	TAs	TAs, PMS, PPM
Bauche 2017	33	NA/1/49/French	LGM, distal limb involvement	p.R111C (homo)	TAs	ND
Bauche 2017	34	NA/6/18/French	LGM, distal limb involvement	p.T392P; p.M499R	TAs	TAs, PMS, PPM
Bauche 2017	35	f/6/16/French	LGM, distal limb involvement, transient ptosis	p.R111C (homo)	TAs	ND
Bauche 2017	36	f/2.5/15/French	LGM, distal limb involvement	p.R111C (homo)	TAs	PMS, PPM
Bauche 2017	37	NA/15/21/French	LGM, distal limb involvement	p.R111H; p.M317L	TAs	ND
Helman 2019	38	M/5/7/Nepalese	LGM, bilateral retinoschisis	p.R14L (homo)	RRF, fiber degeneration	ND
Helman 2019	39	M/0/5/Afghans	Hypotonia, LGM	p.T151K (homo)	RRF, fiber degeneration	ND
Matsumoto 2019	40	F/1.5/38/Japanese	LGM, axial muscle atrophy	c.722_723 insG (homo)	TAs, mild myopathic changes	ND
Luo 2019	41	M/5/23/Chinese	Transient LGM, fatigue	p.K154D; p.D363S	TAs	ND
Szelinger 2020	42	M/0/8/Mexican	Congenital hypotonia, low muscle bulk, dysphagia	p.R230X (homo)	Unspecific myopathic changes	ND
Szelinger 2020	43	M/0/2/Mexican	Intubated and resuscitation	p.R230X (homo)	Necrotizing myopathy, AV	PMS
Ma 2021	44	F/4/15/Chinese	LGM, mild ptosis	p.F5Y; p.F194S	TAs, RV	TAs
Zhao 2021	45	M/0/4/Chinese	Lower limbs weakness	p.R111C; p.A550T	Nonspecific myopathies	ND
Zhao 2021	46	M/6/14/Chinese	LGM	p.V650A (homo)	TAs	ND
Zhao 2021	47	M/5/18/Chinese	LGM	p.T15M	TAs	ND
Zhao 2021	48	M/0/17/Chinese	Upper limbs weakness	p.Y367C; p.G564C	TAs	ND
Zhao 2021	49	F/17/47/Chinese	LGM, bulbar and respiratory involvement	p.R246X; p.T643P	TAs	ND
Zhao 2021	50	M/3/14/Chinese	LGM	p.G26S; p.V291I	Nonspecific myopathies	ND
Zhao 2021	51	M/7/15/Chinese	LGM	p.H677Y	TAs	ND

*Abbreviations*:AAD, age at diagnosis; AAO, age at onset; AV, autophagic vacuoles; EP, endplate; F, female; homo, homozygous mutation; LGM, limb‐girdle muscle weakness; M, male; NA, not available; ND, not done; NMJ, neuromuscular junction; PMS, postsynaptic membrane simplification; PPM, poor postsynaptic membrane; RRF, ragged red fibre; RV, rimmed vacuoles; TAs, tubular aggregates.

Eighteen of 51 patients with muscle biopsy were also examined by electron microscopy. Nine patients showed tubular aggregates on muscle ultrastructure. Extensive autophagic vacuoles were found in two patients. Among the 18 patients, endplate analysis was performed in 12 patients, of which 11 patients revealed significantly reduced and poorly developed junctional fold membrane compared to the normal neuromuscular junction.

## 4 DISCUSSION

In this study, we described two patients with a clinical phenotype of CMS characterized by easy fatigability, progressive limb‐girdle muscle weakness, and response to acetylcholinesterase inhibitor therapy. Electrophysiological assessments revealed positive decrements of repetitive stimulations. Molecular findings indicated biallelic heterozygous mutations in the *GFPT1* gene co‐segregating in the families. Therefore, the two patients were in agreement with the diagnosis of *GFPT1*‐related CMS (Guergueltcheva et al., 2012; Selcen et al., 2013).

Most patients with *GFPT1* mutations present in the first decade with weakness of the more proximal and less distal muscles, and absence of ocular, bulbar and respiratory weakness (Bauché et al., 2017; Guergueltcheva et al., 2012). Like the typical features, the two unrelated patients initially had pronounced fatigability at the early stage of disease, gradually showed limb‐girdle distribution of muscle weakness and decreased therapeutic effectiveness with the disease development. Clinical heterogeneities such as hypotonia, scoliosis, and psychomotor delay were also found in our patients, while no more extra‐muscular symptoms were observed. Accordingly, clinical physicians should carefully make differential diagnosis between the *GFPT1*‐related CMS and myasthenia gravis, metabolic myopathies, or limb‐girdle muscle dystrophy (Witherick & Brady, 2018).

There is a lack of an inherent association between the severity of muscle weakness and the abnormal extent of muscle MRI. The finding of a relatively normal muscle MRI in a patient who showed marked weakness possibly suggested a disorder of neuromuscular junction (Finlayson et al., 2016). This study showed that muscle MRI of *GFPT1*‐related CMS had a tendency of selective distribution of mild fat infiltration characterized by diffusely involving in thigh muscles but sparing of adductor magnus and semimembranosus muscles, as well as diffusely involving in the leg muscles but sparing of medial gastrocnemius. Additionally, the mild hyperintensity in muscles without fat infiltration indicated increased water content. The detailed descriptions about lower limb muscle in patients with *GFPT1*‐related CMS were very rare. Accordingly, it was possible that some *GFPT1*‐related CMS patients might exhibit distinctive muscle MRI, and played an adjunctive role in the diagnosis of CMS, specifically in differentiating CMS from myopathic or dystrophic disorders and between CMS subtypes. Nevertheless, the observations of MRI were limited by a small number of patients, varied age of onset, and different duration of disease.

Although about 70% of *GFPT1*‐related CMS patients showed tubular aggregates that were believed to represent aggregations of misfolded proteins (Schiaffino, 2012), our studies indicated that the pathological changes simultaneously had great diversities. The impairment of neuromuscular junction is a main target due to heavy glycosylation of many important proteins in the neuromuscular junction, while it is possible that *GFPT1* defect could have additional direct pathological effects on extra‐synaptic regions (Hugo & Schlegel, 2017; Niimi et al., 2001). Muscle specimens of patients with hypoglycosylated myasthenia have shown prominent myopathic features including fiber‐type disproportion, degenerating mitochondria, and destruction of the muscle fiber organelles associated with autophagy (Bauché et al., 2017; Guergueltcheva et al., 2012; Helman et al., 2019; Huh et al., 2012; Luo et al., 2019; Ma et al., 2021; Maselli et al., 2014; Matsumoto et al., 2019; O'grady et al., 2016; Selcen et al., 2013; Senderek et al., 2011; Szelinger et al., 2020; Yiş et al., 2017; Zhao et al., 2021). Zebrafish model with GFPT1 knock down also showed abnormalities of both muscle structure and neuromuscular junction (Hugo & Schlegel, 2017). Therefore, it is reasonable that some vacuolar or nonspecific myopathic changes could appear in CMS specimens attributed to *GFPT1*‐related hypoglycosylation of multiple muscle proteins.

It was puzzling that GFPT1 defect in patient one was associated with atypical pathological changes of myofibrillar myopathy (MFM) characterized by desmin deposits, Z‐disc disorganization, and electronic dense granulofilamentous aggregation. More than 200 known glycosyltransferases are responsible for the glycosylation of thousands of proteins in muscle (Zoltowska et al., 2013), of which many MFM‐related proteins, such as desmin, plectin, myotilin, LDB3, and FLNC, should be glycosylated to accomplish physiological functions (Hong et al., 2011). Among these MFM‐related proteins, the plectin crosslinks intermediate filaments to their targets in different tissues, and has been associated with MFM, CMS, and limb‐girdle muscle dystrophy (Winter et al., 2014). In this sense, the underlying hypoglycosylation of plectin that will cause the dysfunction of the protein might be partly in charge of the MFM‐like pathological changes.

The dysfunction of neuromuscular junction is the essence of CMS. The endplates morphology showed that the folds of postsynaptic membrane usually were reduced and simplified, but unspecific abnormalities and even normal endplates could also be observed in *GFPT1*‐related CMS (Zoltowska et al., 2013). Intriguingly, besides the poorly developed endplates, some ring‐like or block‐like materials with electronic dense were observed beneath endplates in our patient. These materials might originate from the disturbance of Z lines or myofibrillar structures. The pathological basis of endplate changes likely stems from hypoglycosylation and altered function of endplate‐specific glycoproteins, such as MUSK, agrin, and dystroglycans (Willems et al., 2016).

In summary, besides the common tubular aggregates, the muscle pathological changes of *GFPT1*‐related CMS also can show rimmed vacuolar myopathy, autophagic vacuolar myopathy, mitochondria‐like myopathy, MFM‐like myopathy, neurogenic features, and unspecific myopathy changes. This extra‐synaptic pathology might be in part responsible for the permanent muscle weakness and resistance to acetylcholinesterase inhibitor therapy. To some extent, the pathological findings might be one of the predictors of the disease outcome.

## CONFLICT OF INTEREST

The authors declare that they have no competing interests.

## AUTHOR CONTRIBUTIONS

Kaiyan Jiang and Yilei Zheng contributed to analysis, interpretation and drafting. Jing Lin contributed to genetic analysis. Yanyan Yu, Xiaobing Li, Xiaorong Wu, and Xin Fang contributed to the acquisition and analysis of data. Meihong Zhou performed the pathological study, Meihong Zhou performed the electrophysiological analysis. Daojun Hong contributed to the study design and revising the manuscript, as well as funding acquisition.

### PEER REVIEW

The peer review history for this article is available at https://publons.com/publon/10.1002/brb3.2469

## Supporting information



Supporting Information

## Data Availability

All relevant data are within the paper and its Supporting Information files.
