# Supplementary material for: Diverse myopathological features in the congenital myasthenia syndrome with GFPT1 mutation
Source: Brain Behav. 2022 Jan 3;12(2):e2469. doi: 10.1002/brb3.2469 (PMC8865156; doi:10.1002/brb3.2469)
Supplement: Supplementary file 1 — Supporting Information [file BRB3-12-e2469-s001.pdf]

Supplemental data

Diverse myopathological features in the congenital myasthenia syndrome with GFPT1 mutation

Kaiyan Jiang[1], Yilei Zheng[1], Jing Lin[1,2], Xiaorong Wu[3], Yanyan Yu[1], Min Zhu[1,2], Xin Fang[1,2], Meihong Zhou[1], Xiaobing Li[1], Daojun Hong[1,2]

1. Department of Neurology, the first affiliated hospital of Nanchang University, Nanchang, 330006, China
2. Department of Medical Genetics, the first affiliated hospital of Nanchang University, Nanchang, 330006, China
3. Department of Ophthalmology, the first affiliated hospital of Nanchang University, Nanchang, 330006, China

Correspondence:

Daojun Hong

Department of Neurology, The First Affiliated Hospital of Nanchang University, Nanchang, 330006, China.

Tel: +86-791-8869-2511; Fax: +86-791-8869- 2511

E-mail: hongdaojun@hotmail.com

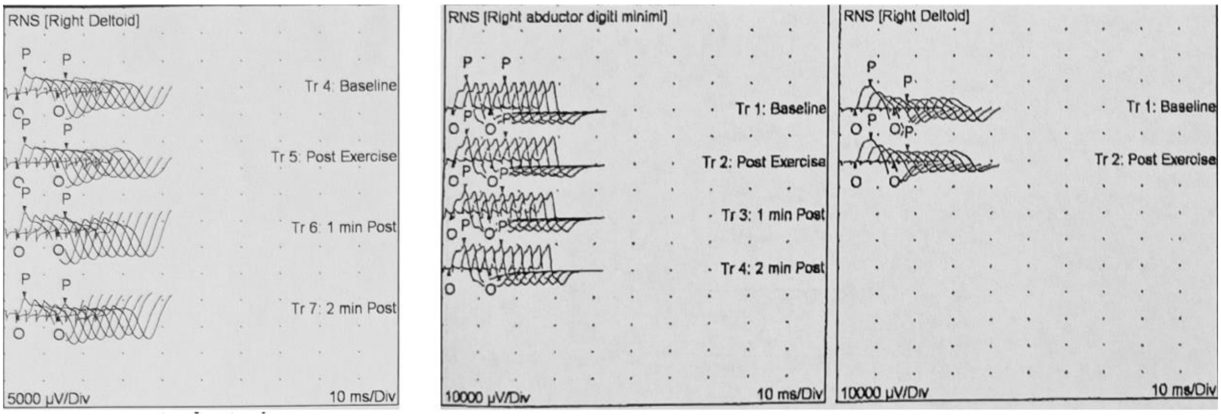

Patient one　　　　　Patient two

Figure S1. Repetitive nerve stimulation (RNS) at 3Hz in patient one revealed positive decrement of compound muscular action potential (CMAP) in the deltoid. RNS at 3Hz in patient two revealed positive decrement of CMAP in the deltoid and abductor digiti minimi muscle.

Table S1. Description of the *GFPT1* gene variants.

Chr	Pos	rsIDs	Ref	Alt	Transcipt Consequence	Protein Consequence	VEP Annotation	Clinical Significance	Allele Freq
2	69590695	rs201322234	G	A	c.331C>T	p.Arg111Cys	missense	Pathogenic (ClinVar)	0.000139211
2	69590694	rs189717232	C	T	c.332G>A	p.Arg111His	missense	Pathogenic (ClinVar)	0.000010625
2	69556825	rs1024585946	G	A	c.1534C>T	p.Arg512Trp	missense	Disease associated-Mutation? (HGMD)	0.000003978

Source gnomAD database v2.1.1 @http://gnomad.broadinstitute.org; http://www.hgmd.org.

Table S2. The clinical summarization of GFPT1-related CMS patients with muscle biopsy.

	Clinical features				CK Elevation x times	EMG		Muscle biopsy TAs	Therapy Response to AChE inhibitors
	Age at onset/ age at report (in years)	Symptom at onset/ motor milestones	Facial/bulbar/respiratory muscle weakness/ other	Limb girdle weakness/fluctuation /course		RNS: decrement at 3Hz%/muscle	Abnormal SFEMG/ Myopathic changes		
P1/m/Iran/ p.D348Y,homozygous	6/31	Muscle weakness, fatigue, worse in summer/ normal	Yes/no/no/ distal limb	Yes/yes/improving after age 20	X 1.5	Yes/ Proximal	ND/ND	Yes	Positive, side effects
P2/f/Turkey/ p.W240X, homozygous	6/26	Muscle weakness, fatigue and pain/normal	None	Yes/yes/ worsening	Normal	14/distal	Yes/yes	Yes	Positive
P3/NA/Libya p.R111C,homozygous	6/23–35	Muscle weakness, fatigue/normal	None	Yes, shoulder **>** pelvic girdle/ND/ND	Normal	65-35/ Trapezius	ND/yes	Yes	Partially positive
P4/m/Spain/ p.M492T and c.*22C **>** A	14/55	Weakness in the upper limbs/ normal	No/no/no/ distal limb	Yes/no/ slight worsening	X1.5	21/deltoid	Yes/yes	Yes	Positive
P5/m/Spain/ p.M492T and c.*22C **>** A	10/50	weakness, falls/normal	No/no/no/ distal limb	Yes/yes/slight Worsening	X1.5	26/deltoid	Yes/yes	Yes	Positive, +3,4 DAP
P6/m/Germany/ p.D43V and p.I121T	5/16	Muscle weakness/ normal	None	Yes/yes/ slight worsening	X2–8	Yes	ND/ND	Yes, on re-revision	Positive
P7/m/UK/ p.R385H and p.R434H	8/23	Fatigue on walk/slight delayed	Yes/no/slight/slight neck and distal limb weakness	Yes, shoulder and pelvic girdle/no/ worsening	X2	Yes/anconeus	Yes/ND	Yes	Positive, +3,4 DAP
P8/m/UK/ p.T15M and p.R496W	6/37	Repeated falls/ normal	Yes/no/no/ distal limb	Yes, shoulder and pelvic girdle/yes/ improving after age 20	X5–10	Yes	Yes/ND	Yes	Positive, +3,4 DAP

	Clinical features				CK Elevation x times	EMG		Muscle biopsy TAs	Therapy Response to AChE inhibitors
	Age at onset/ age at report (in years)	Symptom at onset/ motor milestones	Facial/bulbar/respiratory muscle weakness/ other	Limb girdle weakness/fluctuation /course		RNS: decrement at 3Hz%/muscle	Abnormal SFEMG/ Myopathic changes		
P9/f/Germany/ p.V199F and c.*22 > A	13/26	Fatigue/ normal	None	Yes/yes/ stable	Normal	30-60/ trapezius	ND/yes	Yes, on Revision	Positive
P10/f/Senegal/ p.R512W,homozygous	1/7	Muscle weakness, falls/normal	None	Yes/yes/worsening	Normal	35/ trapezius	ND/yes	Suggestive	Positive, +3,4 DAP
P11/m/Spain/ p.M491T,homozygous	7/19	Muscle weakness/normal	None	Yes/no/stable	Normal	No/distal	ND/yes	One Inclusion	Positive, +3,4 DAP
P12/m/Spain/ c.1278_1281dup and c.*22C > A	1/37	Muscle weakness, falls/slight delayed	No/no/no/ neck and distal muscles	Yes/yes/worsening	Normal	67/ Proximal	Yes/yes	Unspecific myopathic changes	Positive, +3,4 DAP
P13/m/Spain/ c.1278_1281dup and c.*22C > A	First decade/39	Muscle weakness/normal	No/no/no/ neck muscles	Yes/yes/worsening	Normal	14/deltoid	Yes/yes	Yes	NA
P14/m/Italy/ p.T15A and c.621-622del	10/55	Muscle weakness/normal	None	Yes/no/worsening	Slightly	Yes/ trapezius	ND/yes	Yes	Positive
P15/m/Italy/ DNA not available	7/36	Muscle weakness/normal	None	Yes/no/ND	Slightly	Yes/ Proximal	ND/yes	Yes	Positive
P16/m/Sweden/ p.222-223insA and p.R111C	First decade/40	Muscle weakness/ normal	None	Yes/yes/worsening	X12	50/ proximal 20/distal	Yes/yes	Yes	Positive, +3,4 DAP
P17/f/Malta/ p.M491T and c.714_715insA	8/9	Frequent falls, fatigability when walking/ delayed	Learning difficulties	Yes/yes/worsening	Slightly	No/distal	ND/ND	No	Positive at first, but worsening after 2 years of treatment. Improvement with addition of 3,4 DAP
P18/m/Malta/ p.M491T and c.714_715insA	7/13	Difficulties in running, fatigability/normal	Learning difficulties	Yes/yes/worsening	Slightly	ND	ND/ND	No	Positive at first, but effect lost after 3 years of treatment

	Clinical features				CK Elevation x times	EMG		Muscle biopsy TAs	Therapy Response to AChE inhibitors
	Age at onset/ age at report (in years)	Symptom at onset/ motor milestones	Facial/bulbar/respiratory muscle weakness/ other	Limb girdle weakness/fluctuation /course		RNS: decrement at 3Hz%/muscle	Abnormal SFEMG/ Myopathic changes		
P19/m/Korea/ p.E256Q and p.M499T	13/15	Difficulties in climbing stairs and lifting heavy objects, fatigue/ normal	None	Yes/NA/NA	X 1.5	67.1/trapezius 17.1/flexor carpi ulnaris	Yes/yes	Yes	Positive
P20/m/NA/ c.1700-1716dup17 and c.*22C > A	0/16	Poor cry, apneic spells /delayed	No/ no/no/ distal limb	Yes/NA/progressive	NA	Yes	NA/yes	Yes	Positive, +3,4 DAP
P21/f/NA/ p.Arg545Pro and c.*22C > A	8/12	Difficulty climbing steps /NA	No/ no/no/ distal limb	Yes/NA/progressive	NA	Yes	NA/yes	Yes	Positive, +3,4 DAP
P22/f/NA/ c.606-8A>G and c.*22C > A	12/20	Unable to squat/NA	No/ no/no/ distal limb	Yes/NA/progressive	NA	Yes	NA/yes	No	Positive, +3,4 DAP
P23/m/NA/ p.Asp113Gly and p.Met492Thr	19/56	NA/NA	No/ no/no/ distal limb	Yes/NA/progressive	NA	Yes	NA/yes	Yes	Positive, +ephedrine
P24/m/NA/ p.Arg17X and c*22C > A	12/12	Unable to keep up with peers/NA	No/no/yes/ distal limb	Yes/NA/progressive	NA	Yes	NA/yes	No	Positive, +3,4-DAP, albuterol
P25/f/NA/ c.686-2A>G and p.Arg304X	0/1 month	Hypotonia, arthrogryposis, weakness of all except the ocular muscles /NA	Yes/yes/yes/ distal limb	Yes/NA/progressive	NA	Yes	NA/yes	Yes	Limited response to pyridostigmine, 3,4-DAP
P26/m/NA/ p.Arg111Cys, homozygous	10/18	Difficulty climbing steps /NA	No/ no/no/ distal limb	Yes/NA/progressive	NA	Yes	NA/yes	Yes	Positive

	Clinical features				CK Elevation x times	EMG		Muscle biopsy TAs	Therapy Response to AChE inhibitors
	Age at onset/ age at report (in years)	Symptom at onset/ motor milestones	Facial/bulbar/respiratory muscle weakness/ other	Limb girdle weakness/fluctuation /course		RNS: decrement at 3Hz%/muscle	Abnormal SFEMG/ Myopathic changes		
P27/f/NA/ p.T350I and c.1337delA	9/64	Difficulty walking /NA	No/ no/no/ distal limb	Yes/NA/progressive	Mildly elevated	Yes	NA/yes	Yes	Positive
P28/m/NA/ p.Met1fsX2 and p.Thr15Met	4/9	Difficulty walking, clumsy /NA	No/ no/no/ distal limb	Yes/NA/stable	NA	Yes	NA/yes	Yes	Positive, +3,4 DAP
P29/f/USA/ c IVS7-8A>G and c.*22C>A	13/68	Difficulties with running, climbing stairs and keeping her arms up/NA	Yes/no/no/ neck and distal muscles	Yes/NA/NA	Mildly increased	ND	ND/yes	No	Positive, +3,4 DAP
P30/f/ Australia/ c.686-2A>G and p.M358V	0/13	Congenital hypotonia/delayed	Yes/no/no/ contractures, scoliosis	Yes/NA/NA	Normal	Yes	NA	No	Positive
P31/m/Turkey c.686-2A>G, homozygous	13 months/17	Difficulties in rising from the floor/ normal	No/no/no/ neck and distal muscles, mild scoliosis	Yes/NA/NA	X4	NA	NA/yes	No	NA
P32/NA/France / p.Gly39_ Lys75 delinsGlu and p.Arg111His	First decade/68	Muscle weakness, fatigability/ NA	No/no/no/ distal limb	Yes/yes/worsening since 50 years	X 1.5-3	20/trapezius 44/quadriceps 21/abductor digiti minimi 38/tibialis anterior	NA	Yes	Moderate improvement with AChEI and 3,4-DAP
P33/NA/France / p.Arg111Cys, homozygous	1/49	Muscle weakness/NA	No/Mild/No/ distal limb	Yes/yes/worsening since 45 years	X 2	50/trapezius	NA	Yes	The same as P32
P34/NA/France / p.Thr392Pro and p.Met499Arg	6/18	Fatigability/ NA	No/no/no/ distal limb	Yes/yes/ NA	X1.5	52/trapezius 49/ancones 45/tibialis anterior	NA	Yes	The same as P32

	Clinical features				CK Elevation x times	EMG		Muscle biopsy TAs	Therapy Response to AChE inhibitors
	Age at onset/ age at report (in years)	Symptom at onset/ motor milestones	Facial/bulbar/respiratory muscle weakness/ other	Limb girdle weakness/fluctuation /course		RNS: decrement at 3Hz%/muscle	Abnormal SFEMG/ Myopathic changes		
P35/f/France/ p. Arg111Cys, homozygous	6/16	Fatigability/NA	No/no/no/slight transient ptosis, distal limb	Yes/yes/ stable	Normal	34/trapezius 38/ancones 33/tibialis anterior	NA	Yes	The same as P32
P36/f/France/ p.Arg111Cys, homozygous	2.5/15	Muscle weakness/NA	No/no/no/ distal limb	Yes/yes/ worsening then stability in the last 4 years	Normal	50/trapezius 54/ancones 19/abductor digiti minimi 57/tibialis anterior	NA	Yes	Moderate improvement, slight improvement with 3,4-DAP
P37/NA/France / p.Arg111His and p.Met317Leu	15/21	Muscle weakness/NA	No/no/no/ distal limb	Yes/yes/ slight worsening	X2	68/trapezius 28/ancones 40/tibialis anterior	NA	Yes	Moderate improvement with AChEI and 3,4-DAP
P38/m/Nepal/ p.Arg14Leu, homozygous	5/7	Proximal weakness/ basically normal	No/no/no/ bilateral retinoschisis	Yes/NA/NA	Normal	Yes	No/yes	No, maintained fascicular architecture; focal areas of degeneration, regeneration; ragged red fibers	Positive
P39/m/Afghan/ p.Thr151Lys, homozygous	0/5	Delayed walking/delayed	None	Yes/NA/NA	NA	NA	NA	The same as P38	NA
P40/f/Japan/ c.722_723insG, homozygous	1.5/38	Difficulties in walking and running/ normal	None	Yes/yes/worsening	Normal	22.5/right median nerve	No/yes	Yes	Positive
P41/m/China/ p.Lys154Asn and p.Asn363Ser	5/23	Fatigability/NA	None	Yes/yes/ stable	Normal	55/trapezius 62/quadriceps 46/abductor digiti minimi	NA/yes	Yes	Positive
P42/m/Mexica/ p.Arg230Ter,homozygous	0/8	Congenital hypotonia, low muscle bulk, dysphagia requiring gastrostomy/ delayed	Yes/yes/ yes/head and distal muscles	Yes/no/ worsening	NA	14/abductor pollicis minimi 39/trapezius	Yes/NA	No, a population of abnormally small muscle fiber s that was suggestive of a myopathy	NA, negative with 3,4-DAP

	Clinical features				CK Elevation x times	EMG		Muscle biopsy TAs	Therapy Response to AChE inhibitors
	Age at onset/ age at report (in years)	Symptom at onset/ motor milestone	Facial/bulbar/respiratory muscle weakness/ other	Limb girdle weakness/fluctuation /course		RNS: decrement at 3Hz%/muscle	Abnormal SFEMG/ Myopathic changes		
P43/m/Mexica/ p.Arg230Ter,homozygous	0/2 (dead)	Intubated immediately after delivery and required resuscitation with chest compression/dead	Yes/yes/ yes/head and distal muscles	Yes/no/ worsening	NA	NA	NA	No, a mild necrotizing myopathy with extensive autophagic vacuolar pathology	Some benefit
P44/f/China/ p.F5Y and p.F194S	4/15	Frequent falls, difficulties in walking、running and jumping/ normal	No/no/no/ mild bilateral ptosis, distal muscles of the upper limbs	Yes/yes/ worsening at age 11	X2.5	42/left trapezius 50/right trapezius 18/right abductor digiti minimi	Yes/yes	Yes, with rimmed vacuoles	Alleviated partially, improvement with albuterol
P45/m/China/ p.Arg111Cys and p.Ala550Thr	0/4	NA	None	Yes/NA/NA	Normal	Yes	No/no	Mild changes or non-specific myopathies	NA
P46/m/China/ p.Val650Ala, homozygous	6/14	NA	None	Yes/NA/NA	X1.5	Yes	NA	Yes	Positive
P47/m/China/ p.Thr15Met, homozygous	5/18	NA	None	Yes/NA/NA	X2	Yes	NA/yes	Yes	Positive
P47/m/China/ p.Thr15Met, homozygous	5/18	NA	None	Yes/NA/NA	X2	Yes	NA/yes	Yes	Positive
P48/m/China/ p.Tyr367Cys and p.Gly564Cys	0/17	NA	None	Yes/NA/NA	Normal	Yes	NA/yes	Yes	Positive
P49/f/China/ p.Arg246* and p.Thr643Pro	17/47	NA	No/yes/yes /cervical weakness	Yes/NA/NA	Normal	Yes	NA/yes	Yes	Positive
P50/m/China/ p.Gly26Ser and p.Val291Ile	3/14	NA	None	Yes/NA/NA	Normal	Yes	NA/yes	Mild changes or non-specific myopathies	Positive
P51/m/China/ p.His677Tyr	7/15	NA	None	Yes/NA/NA	Normal	Yes	NA	Yes	Positive

The above cases in table S1 came from 14 literatures（Patient 1-18, Patient 19, Patient 20-28, Patient 29 , Patient 30, Patient 31, Patient 32-37, Patient 38-39, Patient 40, Patient 41 , Patient 42-43, Patient 44 , Patient 45-51) ,which are listed in the order of publication time.

Abbreviations: P1: patient 1; LGM: limb-girdle muscle weakness; M: male; F: female; NA: not available; ND: not done; TAs: tubular aggregations; CK: creatine kinase. EMG: electromyography; 3, 4-DAP: 3, 4-Diaminopyridine; RNS: repetitive nerve stimulation; SFEMG: single fiber EMG; AChEI: acetylcholinesterase inhibitors.
